# Digital Store and Forward Imaging as a Quality Assessment Tool for Emergency Plastic Surgery Consultations

**Published:** 2014-01-07

**Authors:** Ian C. Hoppe, Yoomie Lee, Mark S. Granick, Sandra S. Scott

**Affiliations:** ^a^Division of Plastic Surgery, Department of Surgery, Rutgers - New Jersey Medical School, Newark, NJ; ^b^Department of Emergency Medicine, Rutgers - New Jersey Medical School, Newark, NJ

**Keywords:** telemedicine, referral and consultation, surgery, plastic, emergency medicine, patient care

## Abstract

**Introduction:** Plastic surgery is a frequently consulted service in most emergency departments (EDs). The inclusion of a digital image with a plastic surgery consultation would allow the consultant to immediately assess the severity of the condition and either provide instruction to ED staff if minor or mobilize resources to facilitate operative management if more severe. **Methods:** During a 4-month period, all plastic surgery consultations that were seen in person by one of 4 senior plastic surgery residents were included. In addition, an examination of all consultations seen during that time period at the plastic surgery clinic was undertaken to determine the quality of preclinic management. **Results:** During the study period, 78 ED consultations were performed by the plastic surgery residents and included in the study. During the collection period, 374 patients were seen in the plastic surgery clinic. Of these, 154 patients were ED referrals. Evaluation by the senior author revealed that all consultations seen in the ED were appropriate and needed specialist management. Of the ED referrals sent to clinic, but not seen by a plastic surgery consultant in the ED several errors in patient management were noticed. **Conclusions:** The study demonstrated that ED consultations were appropriate for specialty evaluation in all cases, and that insufficient consultations were placed to provide optimal medical care. The role of telemedicine in creating more efficient and effective consultation processes is promising, but numerous legal barriers must be overcome before these modalities can be widely deployed.

Plastic surgery is a frequently consulted service in most emergency departments (EDs). Whether asked to evaluate and treat a simple facial laceration or a complex multiple digit amputation, the plastic surgeon is inextricably linked with the emergency department. Because most plastic surgeons are busy seeing patients in an office or operating, they are notoriously difficult for ED providers to reach. It is therefore important to streamline the interaction between emergency departments and plastic surgery consultants to minimize patient wait times and maximize patient outcomes. The unique nature of plastic surgery emergencies usually makes visual inspection of the patient and relevant radiographs the most important component of subsequent patient management. The inclusion of a digital image with a plastic surgery consultation would allow the consultant to immediately assess the severity of the condition and either provide instruction to ED staff if minor, such as a closed nondisplaced metacarpal fracture, or mobilize resources to facilitate operative management if more severe, such as a digital amputation requiring revascularization.

Telehealth is the process of utilizing communication technology over distance and/or time to facilitate healthcare. This encompasses telemedicine, which involves an interaction between the patient/provider and a consultant separated by distance. This interaction can be synchronous or can be delayed using store and forward technology. Numerous medical specialties already take advantage of telemedicine to improve the delivery of emergency medical care.^1^^-^8 One specialty on the forefront of telemedicine is psychiatry,^9^ with applications including management of school-aged children while in school^10^ to individuals while in prison.^11^ This methodology allows the efficient allocation of limited resources. One study determined that *eConsultation* with digital photographs resulted in similar assessments when compared with on-site evaluations.^12^ Another study concluded that videoconference-based evaluation of wounds in an emergency department setting had high correlation with bedside evaluations in terms of determining severity of wound and need for hospital management.^13^

This study was designed as a Quality Assurance (QA)/Quality Improvement (QI) evaluation to determine the pattern of emergency department plastic surgery consultations and the potential for digital store and forward imaging technology to facilitate the management of plastic surgery consultations. The study was prompted by an incident where the chief of plastic surgery received several complaints. The head of the trauma service complained that it was difficult to get a plastic surgery consultation. The plastic surgery residents complained that they were being consulted for minor injuries not requiring their expertise. The ultimate goal was to improve patient management and outcomes while minimizing the time a patient spends awaiting a consultation in the ED.

## METHODS

During a 4-month period, all plastic surgery consultations that were seen in person by 1 of 4 senior plastic surgery residents were included. Patients seen by the nurse practitioner were not included. De-identified digital images were taken of the injured areas and relevant radiographs. De-identified clinical information about the patients was collected. Following the study period, all images and descriptions were evaluated by the senior author (M.S.G.) to determine the appropriateness of the consultation. It was also determined if one could effectively determine a treatment plan on the basis of the image and brief description. In addition, an examination of all consultations seen during that time period at the plastic surgery clinic was undertaken to determine the quality of preclinic management.

## RESULTS

During the study period, 78 ED consultations were performed by the plastic surgery residents and included in the study. The consultations consisted of 54 hand injuries (14 bony, 25 soft tissue, and 15 complex), 16 head/neck injuries (4 bony, 9 soft tissue, and 3 complex), and 8 other soft tissue injuries (1 leg, 2 trunk, and 5 upper extremity). During the collection period, 374 patients were seen in the plastic surgery clinic. Of these, 154 patients were ED referrals.

**Figure 1:**
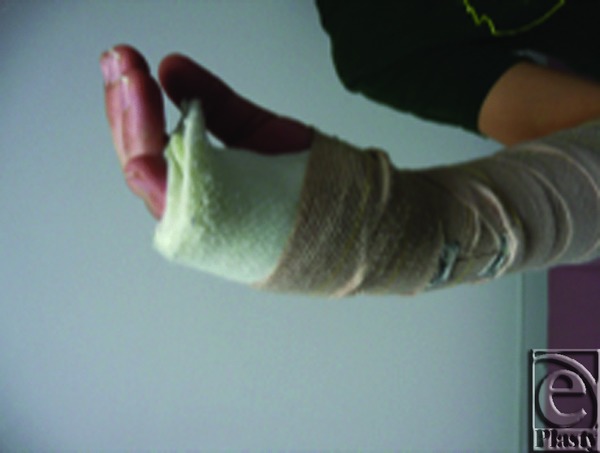
Inappropriate ulnar gutter splint.

**Figure 2:**
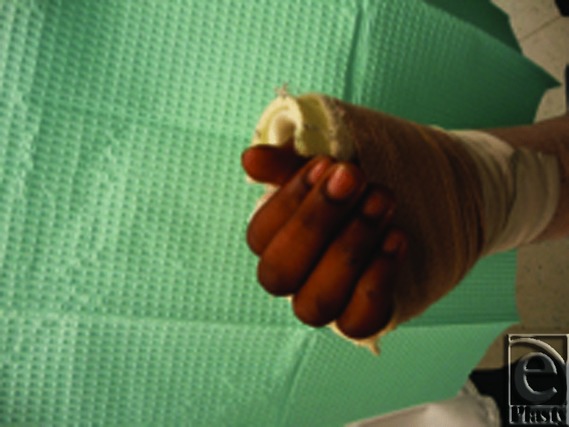
Inappropriate thumb spica splint.

Evaluation by the senior author revealed that all consultations seen in the ED were appropriate and needed specialist management. Of the ED referrals sent to clinic, but not seen by a plastic surgery consultant in the ED, several errors in patient management were noticed. The most common problem encountered with ED referrals not initially managed by plastic surgery was inappropriate splinting (Figs 1-2). In one case, a pediatric patient sustained multiple extensor tendon lacerations and had been given a delayed follow-up appointment following skin suturing and poor positional splinting in the ED.

## DISCUSSION

The QA portion of the study revealed that all of the patients seen in ED consultations required specialty consultation. At the same time, some patients not seen in ED consultation were found to have suboptimal initial care when evaluated in a subsequent specialty clinic. This finding suggests that there should be a higher incidence of ED consultations being initiated. The goal of emergency management of patients with plastic surgical types of injuries is to provide either proper immediate treatment or proper initial management prior to definitive therapy by a specialist. On the basis of the initial complaints that generated this study, the plastic surgery consultation process in the ED requires further study. As it is, the process strains the ability of the plastic surgery service to provide complete coverage.

The QI aspect of this study demonstrated that store and forward digital technology facilitated the evaluation of the plastic surgery consultation process in the ED. In all cases, the severity of the patients’ injuries could be accurately assessed using digital images. The images clearly demonstrated that plastic surgery consultation was appropriate in each case. These findings suggest that there is a role for digital store and forward technology as a QA/QI tool.

There is a strong indication that telehealth may facilitate and improve the plastic surgery consultation process in the ED. Utilizing the same store and forward technology, it might be possible to intervene during the consultation process in a way that accurately evaluates and provides appropriate initial management of the patient. The authors are planning to perform additional QA/QI initiatives to assess the plastic surgery consultation process in greater detail and to evaluate the potential for telehealth intervention.

Several barriers exist to the implementation of a telemedicine process in the current legal environment. The wireless transmission of digital identifiable photographs of patient or any other personal health information (PHI) presents a problem with patient privacy and the Health Insurance Portability and Accountability Act (HIPAA). Measures must be put in place to secure the transmission of PHI in order to ensure that only involved health care professionals have access. A section of HIPAA was specifically created to address the transmission of electronic-PHI (e-PHI) entitled the HIPAA security rule. This requires that covered entities, “1. Ensure the confidentiality, integrity, and availability of all e-PHI they create, receive, maintain or transmit; 2. Identify and protect against reasonably anticipated threats to the security or integrity of the information; 3. Protect against reasonably anticipated, impermissible uses or disclosures; and 4. Ensure compliance by their workforce.”^14^ The language used is intentionally ambiguous to allow for a covered entity to consider its size, complexity, capabilities, infrastructure, costs of security measures, and the impact of potential risks to e-PHI. These regulations have increasingly become a topic of discussion in the plastic surgery literature.^15^

Of particular concern is the use of “smart phones” with the capability to take high-quality digital images. These devices have become relatively ubiquitous with allied health care professionals and are an often-used method of communication between providers. The use of these devices to take on-the-fly pictures of patients presents a problem with regard to patient privacy and the handling and ownership of images once on the electronic device. It is important for institutions to implement guidelines regarding the use of these devices, educate employees, and provide for alternative ways to share information in a HIPAA-compliant manner between practitioners.^16^^,^^17^ Practitioners must also be aware that images taken on “smart phones” may be digitally transmitted to an online back-up service unbeknownst to them, further compromising e-PHI. Other concerns about implementing a telemedicine approach to ED consultations include medical licensing, hospital privileges, medical malpractice, and interstate regulations of telemedical providers. All of these issues must be considered when engaging in a telemedical practice.

## CONCLUSION

A QA/QI evaluation demonstrated the efficacy of store and forward technology in assessing emergency plastic surgical consultations. The study demonstrated that ED consultations were appropriate for specialty evaluation in all cases, and that insufficient consultations were placed to provide optimal medical care. The role of telehealth and telemedicine in creating more efficient and effective consultation processes is promising, but numerous legal barriers must be overcome before these modalities can be widely deployed. Further studies are under way to elucidate this interaction.
